# Increasing trend of fluconazole-non-susceptible *Cryptococcus neoformans* in patients with invasive cryptococcosis: a 12-year longitudinal study

**DOI:** 10.1186/s12879-015-1023-8

**Published:** 2015-07-22

**Authors:** Yi-Chun Chen, Tzu-Yao Chang, Jien-Wei Liu, Fang-Ju Chen, Chun-Chih Chien, Chen-Hsiang Lee, Cheng-Hsien Lu

**Affiliations:** Division of Infectious Diseases, Department of Internal Medicine, Kaohsiung Chang Gung Memorial Hospital, Kaohsiung, Taiwan; Chang Gung University College of Medicine, 123 Ta-Pei Road, Niao-Sung District Kaohsiung City, 833 Taiwan; Department of Laboratory Medicine, Kaohsiung Chang Gung Memorial Hospital, Kaohsiung, Taiwan; Department of Neurology, Kaohsiung Chang Gung Memorial Hospital, 123 Ta-Pei Road, Niao-Sung District Kaohsiung City, 833 Taiwan; Department of Biological Science, National Sun Yat-Sen University, Kaohsiung, Taiwan

**Keywords:** *Cryptococcus neoformans*, Invasive cryptococcosis, Susceptibility, Fluconazole, Risk factors

## Abstract

**Background:**

This study aimed to investigate the rate of fluconazole-non-susceptible *Cryptococcus neoformans* in Southern Taiwan for the period 2001–2012 and analyze the risk factors for acquiring it among patients with invasive cryptococcosis.

**Methods:**

All enrolled strains were isolated from blood or cerebrospinal fluid samples of the included patients. If a patient had multiple positive results for *C. neoformans*, only the first instance was enrolled. Susceptibility testing was performed using the Clinical and Laboratory Standards Institutes M27-A3 broth micro-dilution method. The MIC interpretative criteria for susceptibility to fluconazole were ≤8 μg/ml. A total of 89 patients were included. Patients (*n* = 59) infected by fluconazole-susceptible strains were compared with those (*n* = 30) infected by non-susceptible strains. The patients’ demographic and clinical characteristics were analyzed.

**Results:**

The rate of fluconazole-non-susceptible *C. neoformans* in the study period significantly increased over time (*p* < 0.001). The *C. neoformans* isolated in 2011–2012 (odds ratio: 10.68; 95 % confidence interval: 2.87-39.74; *p* < 0.001) was an independent predictive factor for the acquisition of fluconazole-non-susceptible *C. neoformans*.

**Conclusions:**

The rate of fluconazole-non-susceptible *C. neoformans* has significantly increased recently. Continuous and large-scale anti-fungal susceptibility tests for *C. neoformans* are warranted to confirm this trend.

## Background

*Cryptococcus neoformans* is an encapsulated yeast responsible for life-threatening infections [1]. Pharmacologic management usually consists of primary therapy with amphotericin B, with or without flucytosine, followed by maintenance therapy with fluconazole [2]. Pre-emptive fluconazole therapy for positive serum cryptococcal antigen in patients with human immuno-deficiency virus (HIV) to prevent the development of cryptococcal meningitis is also regarded as cost effective for specific groups [3]. The widespread use of fluconazole for long-term suppressive therapy of cryptococcal infection has become a concern due to the development of fluconazole resistance [4, 5]. Yet despite these concerns, *in vitro* susceptibility testing of *C. neoformans* isolates at the start of therapy is not routinely practiced [2]. Instead, it is reserved for patients with failed primary therapy, those with relapse, or those who develop cryptococcosis and have recent exposure to an anti-fungal drug [2].

In the guidelines of the Infectious Diseases Society of America (IDSA), primary resistance of *C. neoformans* to fluconazole is not a significant clinical problem, as noted in a previous study [6]. In a national surveillance in Taiwan in 2003, only three of 70 (4 %) *C. neoformans* clinical isolates had MICs of fluconazole at concentrations ≥16 μg/ml [7]. Another population-based surveillance in South Africa on *C. neoformans* isolates collected before 2008 still maintained low MIC values to fluconazole [8]. However, the development of microbial resistance is dynamic. The issue of using azoles and *C. neoformans* resistance has been described [9].

A previous study has found that the MIC_90_ of fluconazole against *C. neoformans* increased from 4 to 16 μg/ml in cerebrospinal fluid (CSF) specimens collected between 2001 and 2010 [10]. High rates of *C. neoformans* persistence and frequent relapses have sparked concern for the possible emergence of fluconazole resistance [11]. Increasing *in vitro* resistance to fluconazole in *C. neoformans* isolates has also been proposed [12]. A previous study has elucidated a correlation between fluconazole susceptibility and clinical outcome in patients with cryptococcal meningitis [10]. Thus, an updated surveillance of anti-fungal susceptibility of the clinical strain of *C. neoformans* is desirable to monitor the trend of fluconazole non-susceptible strains [13].

The current study aimed to evaluate the *in vitro* susceptibility of clinical *C. neoformans* isolates against fluconazole between 2001 and 2012 in Southern Taiwan. It also analyzed the risk factors for acquiring fluconazole-non-susceptible *C. neoformans* among patients with invasive cryptococcosis, which was defined as *C. neoformans* escape from the lungs and entering the bloodstream, thereby allowing central nervous system (CNS) dissemination [14].

## Methods

### Study design

*C. neoformans* isolated from patients with invasive cryptococcosis were collected. If the patient had more than one episode of invasive cryptococcosis, only the first episode was included. All enrolled clinical strains were isolated from blood or CSF samples of patients who were admitted to Kaohsiung Chang Gung Memorial Hospital (KCGMH) between January 2001 and December 2012. The KCGMH was a 2700-bed primary care and tertiary referral center in Southern Taiwan. If a patient had multiple positive results for *C. neoformans*, only the first isolate was enrolled for *in vitro* analysis.

This study followed previously published studies and included 46 clinical strains from CSF samples as described previously [10]. To determine the risk factors for acquiring fluconazole-non-susceptible *C. neoformans*, demographic and clinical information were retrieved from medical records retrospectively. The Chang Gung Memorial Hospital’s Institutional Review Board approved the study [No. 102-3819B].

### Data collection and definition

Data on clinical variables included age, sex, and underlying diseases (i.e., AIDS, diabetes mellitus, receiving hemodialysis, chronic kidney disease, liver cirrhosis, chronic lung disease, steroids usage, malignancy, hematologic malignancy, and autoimmune disease). The use of azoles was defined as the intake of fluconazole, itraconazole, voriconazole, or ketoconazole for more than 48 h within three months prior to the first episode of invasive cryptococcosis. Steroid use was defined as the intake of at least 10 mg prednisolone or its equivalent per day for more than two months prior to the infection. The severity of illness at the time of CSF or blood sampling was assessed using the APACHE II scoring method [15] modified as 0 point given to the items PaO_2_ and pH if arterial blood gas analysis was not performed because of the absence of respiratory distress.

Sepsis was defined as a systemic response to infection, manifested by two or more of the following conditions: (1) temperature >38 °C or <36 °C; (2) heart rate >90 beats per minute; (3) respiratory rate >20 breaths per minute or PaCO_2_ < 32 mm Hg; and white blood cell count >12,000 cells/mm^**3**^, <4,000 cells/mm^**3**^, or >10 % immature (band) forms [16]. Septic shock was diagnosed if there was refractory hypotension, signifying that intravenous fluid administration alone was insufficient to maintain adequate blood pressure [16]. The collected laboratory data was on leukocytes, hemoglobin, platelet count, percentage of neutrophils and lymphocytes, and presentation of high titers of CSF and serum cryptococcal antigen defined as more than 1:512 if these data were available.

### Fungal strain

The processing of specimens and identification of isolates were performed by conventional methods using the Vitek Yeast Biochemical Card (BioMerieux, Marcy l’Etoile, France) [17]. The isolated strains were preserved at −70 °C until the experiments.

### Isolation of genomic DNA and PCR amplification

Each strain was grown on SDA plates at 35 °C for two days. Cells were collected and suspended in a TE buffer (100 mM Tris–HCl; pH 8.0, 1 mM EDTA) containing lyticase (Sigma, St. Louis, MO, USA). Glass beads (Sigma) were then added to the micro-tubes and the samples were incubated at 37 °C for 4 h, and mixed in an end-over-end mixer to digest the cell walls. Genomic DNA was extracted from the cells using the High Pure PCR template preparation kit (Roche Applied Science, Mannheim, Germany) according to the manufacturer’s instructions.

The serotype of *C. neoformans* was identified by multiplex PCR. Four primers for cloning laccase gene (*LAC1*) and two for capsule gene (*CAP64*) were used [18]. The *LAC1* differentiated serotypes A, D, B and C and *CAP64* differentiated serotypes D and AD. CNa-70-S and CNa-70-A primer pair (amplified a 695-bp DNA fragment from serotype A), CNa-29-S and CNa-29-A primer pair (amplified a 579-bp fragment from serotype A), CNa-29-S and CNa-70-A primer pair (amplified a 666-bp or a 460-bp fragment from serotype A, 290-bp from serotype B or C) and CNb-49-S and CNb-49-A primer pair (amplified a 448-bp fragment from serotype B or serotype C) were used [19]. The amplified products were separated by electrophoresis and stained with ethidium bromide. The DNA bands were extracted using a gel extraction kit (QIAquick, QIAGEN Sciences, Germantown, MD, USA) and sequenced directly using a BigDye Terminator Cycle-sequencing kit (ABI PRISM 310NT Genomic Analyzer, Perkin-Elmer Applied Biosystems, Foster, CA, USA).

### Fluconazole susceptibility testing

Fluconazole (Pfizer, New York, NY, USA) susceptibilities were determined using the broth micro-dilution method according to the CLSI M27-A3 methodology [20]. Stock solutions were prepared in water and further diluted in RPMI 1640 medium (Sigma) buffered to a pH of 7 with 0.165 M 3-(*N*-morpholino) propanesulfonic acid buffer (Sigma). Aliquots of each agent (0.1 ml) at two-times the concentrations were dispensed into 96-well micro-dilution trays. The yeast inoculum was adjusted to a concentration of 10^4^ CFU/mL before being added to each well. The trays were incubated at 35 °C. The final fluconazole concentrations ranged from 0.25 to 64 μg/ml.

The MICs for fluconazole were the concentrations causing a 50 % reduction in turbidity compared to the growth of the control at 72 h. *Candida krusei* ATCC 6258 and *Candida parapsilosis* ATCC 22019 were used as quality controls. The interpretative criterion for susceptibility to fluconazole was ≤8 μg/ml, as published by the CLSI [20]. The reproducibility of the *in vitro* results was assessed twice on two different days. The geometric mean fluconazole MIC was evaluated each year during the study period.

### Statistical analysis

The annual fluconazole-non-susceptible rate of *C. neoformans* isolated from the patients was calculated. Chi-square test for a linear regression analysis was performed to determine the trends of fluconazole-non-susceptible rates of *C. neoformans* isolated from 2001 to 2012, while simple linear regression was used to estimate the trends of geometric mean fluconazole MIC during the study period. By univariate analysis, continuous variables were expressed as mean ± standard deviation and values were compared by Student’s *t* test. Categorical variables, expressed as numbers and percentages, were compared by chi-square test or Fisher’s exact test, as appropriate.

To identify risk factors for acquiring fluconazole-non-susceptible *C. neoformans*, the patients were categorized as those with fluconazole-non-susceptible (Group 1) or with fluconazole-susceptible (Group 2) *C. neoformans*. Statistically significant variables in univariate analyses between these categories were entered into multivariate analysis using a logistic regression model. Statistical significance was set at a two-tailed *p* < 0.05. Statistical analysis was conducted using the SPSS statistical analysis system (ver.21).

## Results

**Fig. 1:**
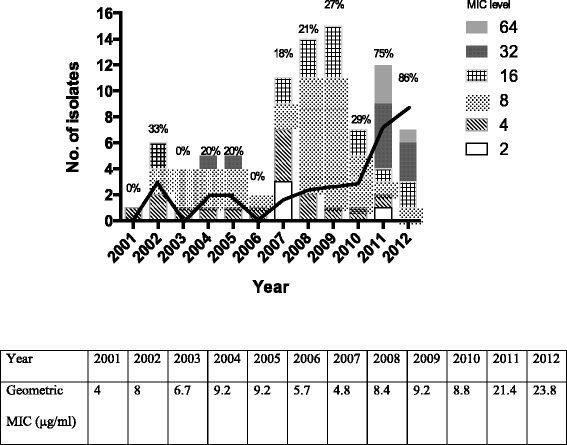
The annual rate (black line) of fluconazole non-susceptible (MICs ≥16 μg/ml) *Cryptococcus neoformans* from 2001 to 2012 significantly increased over time (*p* < 0.001). There was also an increasing trend of geometric mean fluconazole MIC during the study period (*p* < 0.01)

During the study period, 93 isolates of *Cryptococcus* species were identified from blood (n = 48) and CSF (*n* = 45) samples. Eighty-nine isolates were *C. neoformans* var. *grubii* (serotype A) and 4 were *C. gattii* (serotype B). Of the 89 *C. neoformans* isolates, 30 (34 %) were fluconazole-non-susceptible (MICs ≥16 μg/ml). The 89 patients with invasive cryptococcosis were categorized as those who infected by fluconazole non-susceptible *C. neoformans* (Group 1, *n* = 30) and those infected by fluconazole susceptible *C. neoformans* (Group 2, n = 59). The annual rate of fluconazole non-susceptible *C. neoformans* isolated from 2001 to 2012 significantly increased over time (*p* < 0.001). The annual rate of fluconazole non-susceptible *C. neoformans* was 0-33 % in 2001–2006, 18-29 % in 2007–2010, and 75-86 % in 2011–2012 (Fig. 1). There is also a significantly increasing trend over time in the geometric mean fluconazole MIC (*p* < 0.01). Clinical strains cultured in 2011–2012 were specified to clarify the emergence of fluconazole-non-susceptible strains.

The demographic and clinical features and serotypes of *C. neoformans* strains between the two groups were compared (Table 1). Except for the lower proportion of liver cirrhosis in Group 1 (3 % vs. 24 %, *p* = 0.02), the demographic and clinical features were similar in the two groups. Patients in Group 1 had a predominated admission during 2011–2012 (50 % vs. 7 %, *p* < 0.001), previous azole exposure (24 % vs. 7 %, *p* = 0.04), and initial presentation as sepsis (70 % vs. 46 %, *p* = 0.03).

**Table 1:** Risk factors for invasive cryptococcosis caused by fluconazole-non-susceptible *C. neoformans*

Variables	Group 1, *n* = 30 (%)	Group 2, *n* = 59 (%)	*p*
Age	53.8 ± 17.9	58.3 ± 17.8	0.27
Male: female	21:9	37:22	0.50
Admissions 2011-2012	15 (50)	4 (7)	<0.001
Azole exposure^a^	7 (24)	4 (7)	0.04
Co-morbidity			
HIV infection	6/17^b^ (35)	7/36^c^ (19)	0.31
Diabetes mellitus	7 (23)	20 (34)	0.31
Hemodialysis	3 (10)	2 (3)	0.33
Chronic kidney disease	4 (13)	8 (14)	1.00
Liver cirrhosis	1 (3)	14 (24)	0.02
Chronic lung disease	1 (3)	7 (12)	0.26
Steroid user	11 (37)	21 (36)	0.92
Solid cancer	6 (20)	5 (8)	0.17
Hematologic malignancy	3 (10)	4 (7)	0.68
Autoimmune disease	2 (7)	5 (8)	1.00
Disease severity			
APACHE-II score	12.1 ± 8.1	13.9 ± 9.2	0.37
Septic shock	1 (3)	12 (20)	0.05
Sepsis	21 (70)	27 (46)	0.03
Laboratory			
Leukocyte count (1000/mm^3^)	10.46 ± 7.47	9.94 ± 6.66	0.74
Hemoglobin (mg/dL)	11.02 ± 2.16	11.44 ± 2.37	0.43
Platelet count (1000/mm^3^)	156.90 ± 107.89	162.44 ± 98.82	0.81
Neutrophil (%)	78.44 ± 13.40	82.37 ± 12.14	0.18
Lymphocyte (%)	12.84 ± 9.99	10.23 ± 8.33	0.20
Serum cryptococcal Ag >1: 512	11/19^d^ (58)	12/31^e^ (39)	0.19
CSF cryptococcal Ag >1:512	13/20^f^ (65)	16/30^g^ (53)	0.41
Specimen from CSF	15 (50)	30 (51)	1.00

Azole exposure^a^: Patients who received azole therapy (fluconazole, voriconazole, itraconazole, or ketoconazole) for more than 48 h within 3 months prior to the first episode of invasive cryptococcosis

*HIV* human immuno-deficiency virus, *CSF* cerebrospinal fluid, Group 1, patients infected by fluconazole non-susceptible *C. neoformans* (minimal inhibitory concentrations of fluconazole ≥ 16 μg/ml); Group 2, patients infected by fluconazole susceptible *C. neoformans*

^b^9; ^c^37: Numbers of patients with HIV serology test

^d^11; ^e^33: Numbers of patients with serum cryptococcus Ag

^f^11; ^g^32: Numbers of patients with CSF cryptococcus Ag

In multivariate analysis, patient admission in 2011–2012 (odds ratio, OR: 10.68; 95 % confidence interval, CI: 2.87-39.74; *p* < 0.001) was an independent predictive factor for acquiring fluconazole-non-susceptible *C. neoformans* (Table 2).

**Table 2:** Stepwise multiple logistic regression analysis of risk factors associated with invasive cryptococcosis due to fluconazole-non-susceptible *C. neoformans* ^b^

Factors	Comparison	OR (95 % CI)	*p*
Admission during 2011-2012	Yes vs. No	10.68 (2.87-39.74)	<0.001
Liver cirrhosis	Yes vs. No	0.17 (0.02-1.58)	0.12
Initial present with sepsis	Yes vs. No	1.86 (0.62-5.62)	0.27
Azole exposure^a^	Yes vs. No	3.33 (0.73-15.25)	0.12

Azole exposure^a^: patients received azole therapy (fluconazole, voriconazole, itraconazole, or ketoconazole) for more than 48 h within 3 months prior to the first episode of invasive cryptococcosis

*OR* odds ratio, *CI* confidence interval

^b^Statistically significant variables in univariate analyses between these categories were entered into multivariate analysis using a logistic regression model. Statistical significance was set at a two-tailed *p* < 0.05

## Discussion

The current study demonstrated that the rate of fluconazole non-susceptible *C. neoformans* from 2001 to 2012 significantly increased over time (*p* < 0.001). A high fluconazole-non-susceptible rate was especially recognized in the last two years (2011–2012). In the past, the development of secondary resistance to fluconazole during therapy was given more attention than primary resistance. During the 12-year study period, there were 89 *C. neoformans* initial clinical isolates from patients with invasive cryptococcosis, including 30 (34 %) non-susceptible to fluconazole.

The rate of fluconazole non-susceptible *C. neoformans* in the present study is higher than those of previous studies [6, 7, 21]. Thus, non-susceptibility to fluconazole has become a growing problem. A global anti-fungal surveillance study conducted from 1997 to 2007 documents a progressive increase in resistance to fluconazole among *C. neoformans* isolates when results from the time periods 1997 to 2000 (7.3 %), 2001 to 2004 (10.9 %), and 2005 to 2007 (11.7 %) are compared [22]. The problem is especially true among isolates from the Asia-Pacific, Africa/Middle East, and Latin America compared to isolates from Europe or North America [22].

In Asia, most invasive cryptococcosis cases are caused by *C. neoformans* var. *grubii* [23, 24]*.* Similarly, 95.7 % (89/93) of *C. neoformans* isolates from the present study belong to serotype A. One previous study has demonstrated that strains of serotype A are less susceptible to fluconazole than strains of serotype D [21]. Using micro-satellite analysis, there is a different distribution of genotypes of *C. neoformans* var. *grubii* isolates in various countries in Asia, as well as a correlation of the micro-satellite genotypes with the original source of the strain and resistance to anti-fungal agents [25]. Recently, a nationwide multi-center retrospective study in Taiwan has suggested that *C. neoformans* isolates with anti-fungal MIC higher than the epidemiologic cut-off values are rare (one of 203 isolates had fluconazole MIC levels >8 μg/mL) [26, 27]. In Spain, 58 *C. neoformans* clinical isolates were collected from 1990 to 2007. Only 2 strains isolated from HIV patients were fluconazole MIC of 16 μg/ml (3.4 %) [28]. Although the number of fluconazole non-susceptible *C. neoformans* is small according to these two recent studies, the clinical isolates have been obtained before 2010 [27, 28].

In the present study, more patients infected by fluconazole-non-susceptible *C. neoformans* strains experienced azole exposure recently than those infected by fluconazole-susceptible *C. neoformans* strains. The azole exposure in our study is not an independent risk factor for invasive cryptococcosis caused by fluconazole-non-susceptible *C. neoformans* under the multivariate analysis, but the exposure history of our patients may be underestimated because of retrospective study nature. One case series showed that 70 % of patients with fluconazole-resistant cryptococcosis had history of prior exposure to fluconazole [29]. Therefore, the IDSA guidelines for the management of cryptococcal disease recommended *in-vitro* susceptibility testing should be reserved for patients had recent exposure to an antifungal drug [2].

The ability of *C. neoformans* to develop azole resistance is dependent on several mechanisms, including drug target alterations encoded by the gene *ERG11*, which may be obtained through mutations or by over-expression of the gene encoding, over-expression of efflux pumps, and modulation of stress signaling pathways [30]. Moreover, a pattern of cellular responses to the azoles in *C. neoformans* has been reported, as well as the term hetero-resistance, which occurs when a single cell gives rise to a progeny with heterogeneous resistance phenotypes, even with a small subset of progeny that are highly resistant to azole [31, 30]. The resistant sub-population can adapt to increasing concentrations of azoles in a stepwise manner [30]. The formation of disomic chromosomes in response to fluconazole stress is closely associated to *ERG11* and *AFR1,* the major transporter of azoles in *C. neoformans* in both serotypes A and D [32]. That is an adaptive mechanism in *C. neoformans* that plays an important role in the failure of fluconazole therapy on cryptococcosis [32].

Pan et al. report that fluconazole has the broadest (0.125-32 μg/mL) and the highest MIC value, and lowest activity (MIC_90_ = 4 μg/mL) against *C. neoformans* compared to other azoles like intraconazole, voriconazole, posaconazole, and isavuconazole. These new generation triazoles may become an important therapeutic choice to currently used anti-fungals [25].

This study still has several limitations. It is a single center study. Differences in fluconazole-susceptibility rates may exist owing to geographical variations. The retrospective use of patient medical record usually means that some data are missing or misclassified and not all of the patients have been checked for HIV. The number of patients or duration of azole exposure may be underestimated. In addition, drug exposure has not been quantified definitely. These factors may yield more conservative results. This 12-years longitudinal study continuously investigates the susceptibility of *C. neoformans* against fluconazole. In the last two years, there has been increasing fluconazole non-susceptibility. The possibility of a clonal phenomenon associated with fluconazole non-susceptibility is doubtful. However, all patients with invasive cryptococcosis have been diagnosed sporadically for the study period (2001–2012). There is no relationship to admission date and inhabited area among these patients. Further studies with genotyping by pulsed field gel electrophoresis and mating type are needed*.* Nevertheless, the results here serve to remind clinicians that primary resistance to fluconazole of *C. neoformans* may not be persistently low.

## Conclusions

This study reveals that the fluconazole non-susceptibility of *C. neoformans* has increased in the last two years of this 12-year longitudinal study. Continuous and large-scale anti-fungal susceptibility tests for *C. neoformans* are necessary to confirm this trend.

## Abbreviations

CLSI: Clinical and laboratory standards institutes
MIC: Minimal inhibitory concentration
HIV: Human immune-deficiency virus
IDSA: Infectious disease society of America
CSF: Cerebrospinal fluid
CNS: Central venous system
KCGMH: Kaohsiung Chang Gung Memorial Hospital
AIDS: Acquired immunodeficiency syndrome
APACHE II: Acute physiology and chronic health evaluation II
PaO2: Partial pressure of oxygen in arterial blood
PaCO_2_: Partial pressure of carbon dioxide in arterial blood
PCR: Polymerase chain reaction
DNA: Deoxyribonucleic acid
SDA: Sabouraud dextrose agar
RPMI: Roswell Park Memorial Institute
